# Serum 8,12-iso-iPF2α-VI Isoprostane Marker of Oxidative Damage and Cognition Deficits in Children with Konzo

**DOI:** 10.1371/journal.pone.0107191

**Published:** 2014-09-15

**Authors:** Bumoko G. Makila-Mabe, Kambale J. Kikandau, Thérèse M. Sombo, Daniel L. Okitundu, Jean-Claude Mwanza, Michael J. Boivin, Mumba D. Ngoyi, Jean-Jacques T. Muyembe, Jean-Pierre Banea, Gerard R. Boss, Desiré Tshala-Katumbay

**Affiliations:** 1 Department of Neurology, University of Kinshasa, Kinshasa, Congo-Kinshasa; 2 Department of Biomedical Sciences, University of Kinshasa, Kinshasa, Congo-Kinshasa; 3 Department of Ophthalmology, University of North Carolina at Chapel Hill, Chapel Hill, North Carolina, United States of America; 4 Department of Psychiatry and Neurology/Ophthalmology, Michigan State University, East Lansing, Michigan, United States of America; 5 Department of Tropical Medicine, University of Kinshasa, Kinshasa, Congo-Kinshasa; 6 Department of Parasitology, Institut National de Recherches Biomédicales, Kinshasa, Congo-Kinshasa; 7 Department of Nutrition, School of Public Health & National Nutrition Program, Ministry of Health, Kinshasa, Congo-Kinshasa; 8 Department of Medicine, University of California San Diego, La Jolla, California, United States of America; 9 Department of Neurology, Oregon Health & Science University, Portland, Oregon, United States of America; 10 Oregon Institute of Occupational Health Sciences, Oregon Health & Science University, Portland, Oregon, United States of America; Vanderbilt University Medical Center, United States of America

## Abstract

We sought to determine whether motor and cognitive deficits associated with cassava (food) cyanogenic poisoning were associated with high concentrations of F2-isoprostanes, well-established indicators of oxidative damage. Concentrations of serum F2-isoprostanes were quantified by LC-MS/MS and anchored to measures of motor proficiency and cognitive performance, which were respectively assessed through BOT-2 (Bruininks/Oseretsky Test, 2^nd^ Edition) and KABC-II (Kaufman Assessment Battery for Children, 2^nd^ edition) testing of 40 Congolese children (21 with konzo and 19 presumably healthy controls, overall mean age (SD): 9.3 (3.2) years). Exposure to cyanide was ascertained by concentrations of its main metabolite thiocyanate (SCN) in plasma and urine. Overall, SCN concentrations ranged from 91 to 325 and 172 to 1032 µmol/l in plasma and urine, respectively. Serum isoprostanes ranged from 0.1 to 0.8 (Isoprostane-III), 0.8 to 8.3 (total Isoprostane-III), 0.1 to 1.5 (Isoprostane-VI), 2.0 to 9.0 (total Isoprostane-VI), or 0.2 to 1.3 ng/ml (8,12-iso-iPF2**α**-VI isoprostane). Children with konzo poorly performed at the BOT-2 and KABC-II testing relative to presumably healthy children (p<0.01). Within regression models adjusting for age, gender, motor proficiency, and other biochemical variables, 8,12-iso-iPF2**α**-VI isoprostane was significantly associated with the overall cognitive performance (β = −32.36 (95% CI: −51.59 to −13.03; P<0.001). This model explained over 85% of variation of the KABC-II score in children with konzo, but was not significant in explaining the motor proficiency impairment. These findings suggest that cognitive deficits and, possibly, brain injury associated with cassava poisoning is mediated in part by oxidative damage in children with konzo. 8,12-iso-iPF2**α**-VI isoprostane appears to be a good marker of the neuropathogenic mechanisms of konzo and may be used to monitor the impact of interventional trials to prevent the neurotoxic effects of cassava cyanogenic poisoning.

## Introduction

Chronic reliance on cyanogenic cassava (a.k.a manioc or tapioca) as staple food and poor nutrition have been incriminated in the etiology of konzo, tropical ataxic neuropathy (TAN), and, reportedly, a motor neuron-cerebellar-parkinson-dementia syndrome (MNCPD) among rural populations of sub-Saharan Africa [1]–[7]. In contrast to TAN and MNCPD, konzo still occurs in sub-Saharan Africa [8]–[12]. We recently showed that older children, females, and those undernourished were at higher risk for motor and cognitive deficits among populations that rely almost exclusively on cassava as staple diet [9], [13]. While there is a substantial body of knowledge on the markers of susceptibility to cassava cyanogenic poisoning and related neurological deficits, little is known on the biomarkers of the neuropathological processes, potential targets of efforts to prevent the development of the aforementioned deficits once the children have been exposed to cyanogenic compounds arising from consumption of poorly processed cyanogenic cassava [5].

Previous studies including experimental modeling of cyanide poisoning have suggested that oxidative damage may play a central role in the pathogenesis of cassava-associated neurological deficits [14]–[16]. Such indication has been lacking for *in-vivo* human studies on konzo, a condition with a myriad of etiological factors with a potential to trigger a massive oxidative response. These include but not limited to the direct toxicity of cyanide on mitochondria, a major source of reactive oxygen species (ROS), which can cause oxidative damage and cell death [5]. Markers of oxidative damage include oxidation by-products of proteins, lipids, and nucleic acids [17]. The Biomarkers of Oxidative Stress (BOSS) Study, an independent and multi-investigator study sponsored by the National Institute of Environmental Health Sciences has determined that the most accurate method to assess *in vivo* oxidative stress status is to quantify plasma or urinary free isoprostanes (F2-IsoPs), which are by-products of non-enzymatic oxidative damage of lipids [18]–[20]. In this study, we sought to determine whether the motor and cognitive deficits associated with cassava (food) cyanogenic poisoning were associated with high concentrations of F2-IsoPs.

## Subjects and Methods

### Subjects

Forty subjects including 17 with the severe form of konzo and two pairs of twins with mild konzo and 19 presumably healthy controls (overall mean (SD) age: 9.2 (3.0) years) from a previously investigated outbreak area were included in the present biomarker study [9]. Children with the diagnosis of konzo have fulfilled the WHO criteria for the disease i.e. presenting with a visible symmetric spastic abnormality of gait while walking or running; a history of onset of less than 1 week followed by a non-progressive course in a formerly healthy person; and bilaterally exaggerated knee or ankle jerks without signs of disease of the spine. The severity of the disease was graded as follows: mild case  =  able to walk; moderate case  =  need support (one or two sticks) to walk, severe case  =  unable to walk [21]. Children with history of illness that may affect the CNS (e.g., cerebral malaria, HIV I/II, or HTLV-I/II infections) were excluded. All study participants were subjected to neuropsychological testing using the Kaufman Assessment Battery for Children, 2^nd^ edition (KABC-II) for cognition and the Bruininks/Oseretsky Test, 2^nd^ Edition (BOT-2) measure for motor proficiency as previously described [9].

#### Ethics statement

Informed consent and child assent were obtained verbally by investigators who were fluent in Lingala and/or Kikongo, the local spoken languages. Parents who allowed their children to participate in the study were then asked to sign on the consent forms that were kept for records at the study office. Ethical approval of research activities including informed consent and assent procedures was obtained from the Oregon Health & Science University (OHSU) Institutional Review Board FWA00000161 and from the Ministry of Health of the Democratic Republic of Congo (DRC).

### Sample collection and biochemical assays

A team of trained laboratory technicians collected samples from people living in remote and rural Kahemba district (DRC). Interviews with members of a technical team from the Ministry of Health have revealed that shortcuts in cassava processing times were common in Kahemba. In this area, the analysis of flour samples from 18 consenting households revealed cyanide concentrations ranging from 30 to 200 ppm, well-above the 10 ppm safe limit proposed by the World Health Organization (Joint FA0/OMS report on food contaminants, Rotterdam, 2009). In study subjects, blood was collected through venipuncture in Vacutainer tubes with no anticoagulants and kept at room temperature for approximately 2 hours. The blood was centrifuged at 15,000 rpm for 15 min, and the serum was aliquoted in cryotubes and flash-frozen in liquid nitrogen. The serum was then shipped to Kinshasa, the capital city of DRC, and stored at −80°C until shipment to OHSU on dry ice for biochemical analyses. One-time on-spot urine collections were carried out at the time of the study. Samples were also immediately flash-frozen in liquid nitrogen, shipped to Kinshasa, and stored at −80°C until use for biochemical analyses. Serum albumin, urea, creatinine, and lipids were determined using a piccolo automated system (Abaxis, Germany). Exposure to cyanogenic compounds was ascertained by measuring urinary SCN concentrations using a previously described semi-quantitative method and plasma SCN concentrations using a modified version of a previously published protocol [22], [23].

### LC-MS/MS for Free Isoprostanes

The initiation of lipid peroxidation of polyunsaturated fatty acids in phospholipid produces a series of products known as F2-Isops initially described by Morrow and coworkers [24]. Four different regioisomers of the F_2_-IsoPs are produced, each of which is comprised of eight racemic diastereomers [24]. These prostaglandin-like metabolites are produced non-enzymatically while arachidonic acid esterifies in lipid membranes. The nomenclature that will be used refers to these products as class III, IV, V or VI [25]. Standards currently commercially available use the same nomenclature (Cayman, Ann Arbor, MI).

#### Sample preparation for serum free isoprostanes

We used a slightly modified solid phase extraction described by Zhao et al. [26]. Briefly, aliquots of serum (0.5 ml) were transferred to tubes containing butylated hydroxytoluene (BHT) at a final concentration of 20 mM and d_4_-iPF_2α_-III and d_4_-iPF_2α_-VI (1 ng as the internal standards) were added to each sample. Each sample was acidified with 1 ml of 1 N HCl and 2 ml of 100 mM formate buffer (pH 3.0), and centrifuged at 2000 *g* for 10 min and the supernatant submitted to solid-phase extraction.

The sample was then applied to a 60 mg Oasis HLB extraction cartridge preconditioned with 1 ml of methanol and 2 ml of 10 mM formate buffer (pH 3.0). The cartridge was washed with 3 ml of the formate buffer followed by 3 ml of acetonitrile:water (15∶85 v/v) and then 3 ml of 9∶1 (v/v) hexane:ethyl acetate. After drying the column for 10 min under vacuum, the F_2_-IsoPs were eluted by washing the cartridge with 6 ml of hexane–ethyl acetate–2-propanol (30∶65∶5 by volume). The eluates were dried under reduced pressure, dissolved in HPLC solvent (30% acetonitrile containing 0.05% acetic acid) and then analyzed with LC-MS/MS as described below.

#### Mass spectral parameters

LC-MS/MS was conducted using an ABSciex 5500 QTRAP. In preliminary studies we used commercially available standards and found that each of the four classes generated abundant molecular ions at *m/z* = 353 under electrospray ionization (ESI) conditions in negative mode. Using direct infusion, optimal instrument parameters were obtained and the product ion spectra of each of the four classes determined (**Table 1**). We confirmed that unique product ions were produced for each of the four classes. They were iPF_2α_-III, *m/z* = 353→193; iPF_2α_-IV, *m/z* = 353→127; iPF_2α_-V, *m/z* = 353→151, and iPF_2α_-VI, *m/z* = 353→115. In addition, PGF_2α_ had a transition of *m/z* = 353→193. Tetradeuterated internal standards for iPF_2α_-III, iPF_2α_-VI, and PGF_2α_ were monitored with *m/z* = 357→197 for d_4_-iPF_2α_-III and d_4_-PGF_2α_, while d_4_- iPF_2α_-VI was monitored at *m/z* = 357→115 since the deuterium was not in the fragment ion. The findings were consistent with the literature [27].

**Table 1:** Optimized MRM parameters.

Compound	Q1	Q3	DP	CE	CXP	DWELL	INT STD
iPF_2α_-III	353	193	−105	−36	−3	25 ms	iPF_2α_-d_4_-III
iPF_2α_-d_4_-III	357	197	−125	−36	−7	25 ms	n/a
iPF_2α_-VI	353	115	−55	−30	−5	25 ms	iPF_2α_-d_4_-VI
iPF_2α_-d_4_-VI	357	115	−125	−30	−9	25 ms	n/a
*ent*-PGF2α	353	309	−55	−28	−5	25 ms	d_4_- PGF_2α_
d_4_- PGF2α	357	313	−115	−30	−13	25 ms	n/a
iPF_2α_IV	353	125	−75	−32	−11	25 ms	iPF_2α_-d_4_-VI
iPF_2α_ 8,12-VI	353	115	−85	−30	−7	25 ms	iPF_2α_-d_4_-VI

Q1  =  1st Quadrupole setting; Q3  =  3^rd^ Quadrupole setting; DP  =  Declustering Potential; CE  =  Collision Energy; CXP  =  Collision Cell Exit Potential; DWELL  =  Dwelling time; INT STD  =  Internal Standard.

Analysis of the extracted samples and standards was accomplished using a Shimadzu Prominence LC system interfaced to the 5500 QTRAP. Both systems are controlled using Analyst version 1.51 software. The LC method used a 100×2.0 mm 3 µm BetaBasic-18 column preceded by a 2×10 mm guard column of the same material. The mobile phase consisted of A (0.05% acetic acid in water) and B (0.05% acetic acid in acetonitrile) with the following gradient: 30% B at 0 min for 0.1 min; to 40% B in 1 min; to 45% B in 4 min; to 60% B in 5 min; to 62.5% B in 9 min; to 95% B in 5 min and hold for 2 min then re-equilibrate to 30% B for 6 min. The flow rate was 0.50 ml/min and the column oven at 40°C. The source parameters were: voltage, −4000 V; temperature, 450°C; curtain gas 40; gas1 and gas2, 40; exit potential, −10 and CAD gas, high. The instrument was operated in the MRM mode using the transitions described in Table 1. Quantification was done using Analyst 1.5 software comparing the peak area of the d_4_-internal standards to known standards (from 20 pg/ml to 10 ng/ml) for each of the four classes of isoprostanes. The total area of the peaks obtained for each unique transition was used to assess the concentrations of each of the four classes as described [28]. The extracted ion current we observed for iPF_2α_-III and iPF_2α_-VI from plasma were consistent with the literature where the iPF_2α_-III, *m/z* = 353→193 transitions reveals the presence of a large number of IsoP peaks representing various diastereomers [28], [29]. Significant concentrations of iPF_2α_-III, iPF_2α_-VI, and iPF2_α_ 8, 12-VI were observed and are reported.

### Statistical analyses

The BOT-2 score was used as outcome measure of motor proficiency and the KABC-II global mental processing index, herein referred to as KABC-II score, as a measure of cognitive performance. Mean scores or biochemical measures were compared using Student *t* test. Spearman coefficients were computed for correlations between biochemical and neurological measures of interest. Linear regression was used to explore the association between BOT-2 or KABC-II scores and demographic (age, sex) as well as biochemical characteristics. The regression models initially examined each explanatory variable separately for association with mean BOT-2 and KABC-II scores, with regression parameters providing crude (unadjusted) estimates of the associations. Subsequent multivariable models were constructed using age and sex as balancing variables (for all models) with additional terms added based on initial univariable findings. Interactions were examined to determine whether sex modified any of the variables retained in the multivariable model. Standard checks for model adequacy (Shapiro-Wilk test and Q-Q plot of residuals, plots of residuals against fitted values, screening for outliers/high leverage) were performed on the baseline models. The baseline models were then supplemented with measures of isoprostanes (separately) to determine whether either measure could further improve performance with and without adjustment for concentration of triglycerides. The Bayesian Information Criterion (BIC) was used to compare the non-nested models to determine which additional predictor was a more potent supplement to the baseline models. Predictors from the best models were then used to build konzo-specific models of association. Stata (version 11.2) was used for all analyses with significance level set at 0.05.

## Results

### Neurological impairments and biochemical profiles

**Figure 1:**
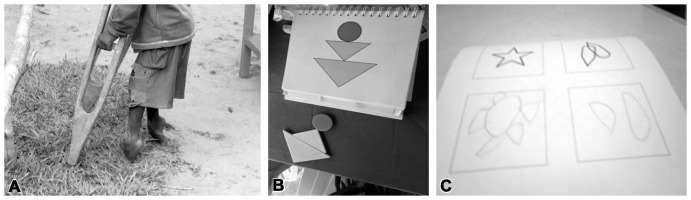
Illustrative example of neurodisability in konzo. (A) Spastic stance in a boy severely affected by konzo. (B) Depiction of deficits at KABC-II cognition testing. (C) Deficits in fine motor control at the BOT-2 testing.

Physical examination and neuropsychological testing revealed overt signs of motor dysfunction and spasticity as well as signs of cognitive impairment and deficits in fine motor control (**Figure 1**).

Concentrations of plasma and/or urinary SCN confirmed that all study subjects were exposed to cyanogenic compounds [6] (**Table 2**). Overall, SCN concentrations ranged from 91 to 325 and 172 to 1032 µmol/l in plasma and urine, respectively. Children with konzo had low mean concentrations of serum albumin and unadjusted plasma SCN, the main metabolite of cyanide, relative to those with no konzo (p≤0.05). After adjusting for albumin concentrations, children with konzo had higher plasma SCN per unit of protein relative to those with no konzo. The difference was, however, not significant (p>0.10). Children with konzo also had higher concentrations of triglycerides relative to those with no konzo (p = 0.02).

**Table 2:** Age, biochemical measures and neurocognitive proficiency scores by konzo status.

Characteristics, Units (Konzo/Non-Konzo ratio)	Konzo Children Mean (SD)	Non-Konzo Children Mean (SD)	*p*-Value
Age, Years (21/19)	9.2 (3.2)	9.3 (2.9)	0.94
Duration of konzo, Months (21/0)	28.0 (20.2)		
Plasma SCN, µmol/l (13/13)	177.8 (61.5)	229.7 (66.8)	0.05
Urinary SCN, µmol/l (19/12)	434.5 (265.0)	387.0 (195.8)	0.59
Serum albumin, µmol/l (20/15)	469.9 (116.1)	606.8 (61.5)	<0.01
Triglycerides, mmol/l (20/15)	1.5 (1.1)	1. (0.2)	0.02
Total lipids, g/l (20/15)	4.7 (2.1)	3.9 (0.9)	0.08
Isoprostane-III, ng/m (20/15)	0.3 (0.1)	0.3 (0.1)	0.68
Total Isoprostane-III, ng/ml (20/15)	3.1 (1.7)	3.4 (2.2)	0.64
Isoprostane-VI, ng/ml (20/15)	0.3 (0.3)	0.2 (0.1)	0.09
Total Isoprostane-VI, ng/ml (20/15)	3.2 (1.4)	2.9 (0.7)	0.47
8,12-iso-iPF2alpha-VI isoprostane, ng/ml (20/15)	0.5 (0.2)	0.3 (0.1)	0.04
BOT-2 motor score (21/16)	21.2 (2.3)	35.8 (7.0)	<0.01
KABC-II cognition score (21/16)	53.5 (6.8)	61.8 (10.0)	<0.01

Children with konzo had low concentrations of serum albumin, plasma SCN, and neurology proficiency scores. They had, however, higher concentrations of serum triglycerides and 8,12-iso-iPF2**α**-VI isoprostane relative to those with no konzo.

Overall, the concentrations of serum isoprostanes ranged from 01 to 0.8 ng/ml for free isoprostane-III, 0.8 to 8.3 ng/ml for total isoprostane-III, 0.1 to 1.5 ng/ml for free isoprostane-VI, 2.0 to 9.0 ng/ml for total Isoprostane-VI, and 0.2 to 1.3 ng/ml for free 8,12-iso-iPF2**α**-VI isoprostane. Children with konzo had higher mean serum concentrations of 8,12-iso-iPF2**α**-VI isoprostane relative to those with no konzo (p = 0.04). No significant difference was found across the two study groups with respect to mean concentrations of the other forms of isoprostanes (p≥0.09). A significant difference was observed in BOT-2 and KABC-II scores between konzo and non-konzo children (p<0.01) (**Table 2**).

With respect to the severity of paralysis, the unadjusted mean (SD) plasma SCN concentration was 177. 84 (61.46) in children with the severe form of konzo relative to 229.69 (66.76) µmol/l relative to those with no konzo (p = 0.05). After adjusting for albumin concentrations, children with severe konzo had higher plasma SCN per unit of protein relative to those with no konzo. The difference was, however, not significant (p = 0.37). The mean (SD) free 8,12-iso-iPF2**α**-VI isoprostane was 0. 52 (0.27) in children with the severe form of konzo relative to 0.35 (0.08) ng/ml relative to those with no konzo (p = 0.09). Comparisons of mean concentrations of other forms of isoprostanes across groups with different degree of severity showed no significant differences (all p>0.45). The two pairs of twins with the mild form of konzo had motor proficiency and cognitive performance scores as well as main biochemical characteristics not remarkably different from the remaining study subjects. We found no significant differences in serum concentrations of isoprostanes between males and females except for the mean concentration of total isoprostane-VI, which appeared to be higher in females relative to males (p = 0.04) (**Table 3**).

**Table 3:** Gender-specific concentrations of serum isoprostanes (ng/ml).

Compound	Male (N = 20) Mean (SD)	Female (N = 15) Mean (SD)	p-Value
Isoprostane-III	0.3 (0.1)	0.3 (0.2)	0.44
Total Isoprostane-III	3.2 (1.8)	3.2 (2.1)	0.99
Isoprostane-VI	0.3 (0.1)	0.4 (0.3)	0.26
Total Isoprostane-VI	2.8 (0.6)	3.5 (1.6)	0.04
8,12-iso-iPF2**α**-VI isoprostane	0.4 (0.1)	0.5 (0.3)	0.07

Females appeared to have higher concentrations of total isoprostane-VI relative to males.

### Correlations between concentrations of serum isoprostanes, triglycerides, albumin, and neurocognitive proficiency scores

**Figure 2:**
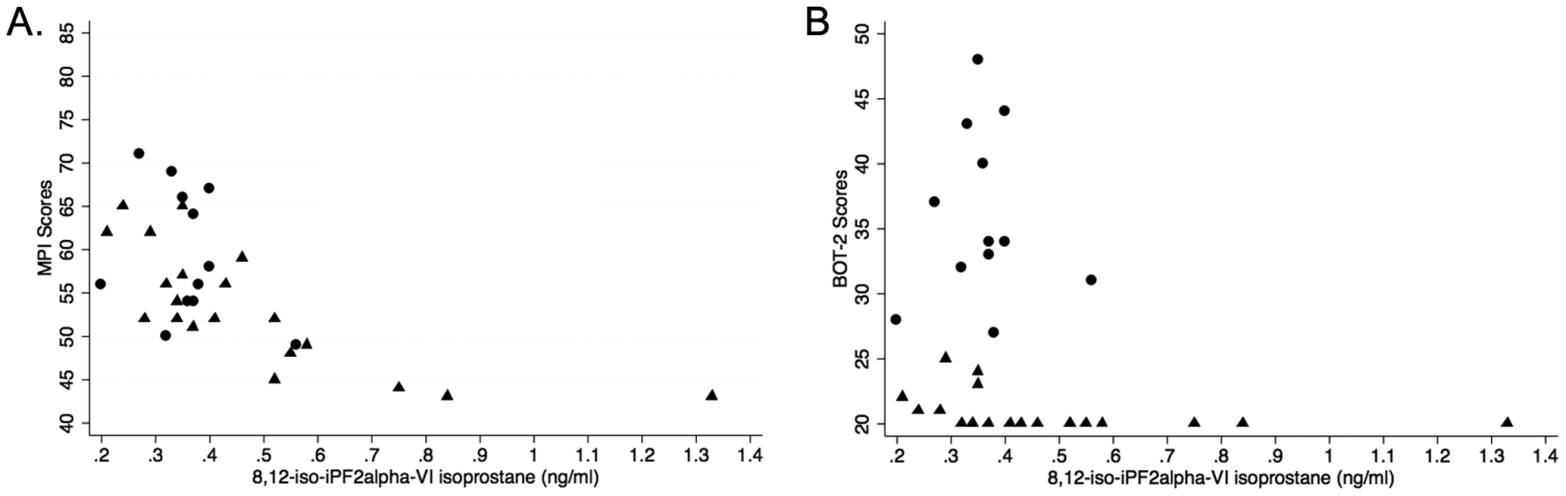
Correlations between motor/cognition performance scores and levels of serum isoprostanes. Low motor or cognition performance significantly correlated with high concentrations of 8,12-iso-iPF2alpha-VI isoprostane in children affected by konzo. (A) MPI (mental processing index) also referred to as KABC-II scores in main text versus serum level of 8,12-iso-iPF2α-VI isoprostane (triangles  =  konzo children, r = −0.78, p = 0.00; circles  =  non-konzo children, r = −0.24, p = 0.47). (B) BOT-2 scores versus serum level of 8,12-iso-iPF2α-VI isoprostane (triangles  =  konzo children, r = −0.63, p<0.01; circles  =  non-konzo children, r = −0.06, p = 0.86).

A significant correlation was found between concentrations of various forms of isoprostanes (all p<0.01) except for isoprostane-III and total isoprostane-III, which showed no correlation with concentrations of 8,12-iso-iPF2**α**-VI isoprostane (0.06≤p≤0.1). Significant correlations were found between concentrations of isoprostane-VI, total isoprostane-VI, and/or 8,12-iso-iPF2**α**-VI isoprostane, and concentrations of serum triglycerides, albumin, BOT-scores, or KABC-II scores. In particular, strong positive correlations were found between concentrations of isoprostane-VI, total isoprostane-VI, 8,12-iso-iPF2**α**-VI isoprostane, and triglycerides (p = <0.01); or between serum concentrations of albumin and neurology proficiency scores (p≤0.04). In contrast, serum concentrations of 8,12-iso-iPF2**α**-VI isoprostane negatively correlated with concentrations of albumin and neurocognitive proficiency scores (p≤0.02) (**Table 4** **and** **Figure 2**). The duration of the disease, the KABC-II or BOT-2 scores, or the concentrations of albumin or F2-isoprostane isomers did not correlate with concentrations of plasma or urinary SCN (all p>0.10).

**Table 4:** Spearman coefficients (p-value) for correlations between concentrations of isoprostanes, triglycerides, albumin, and neurocognitive proficiency scores (N = 32).

Parameter	Isoprostane-VI	Total Isoprostane-VI	8,12-iso-iPF2α-VI Isoprostane	Serum Triglycerides	Serum Albumin	BOT2-Scores
Isoprostane-VI						
Total Isoprostane-VI	0.56 (0.00)					
8,12-iso-iPF2**α**-VI Isoprostane	0.57 (0.00)	0.73 (0.00)				
Serum Triglycerides	0.26 (0.14)	0.18 (0.31)	0.45 (0.00)			
Serum Albumin	−0.21 (0.24)	−0.19 (0.28)	−0.40 (0.02)	−0.40 (0.03)		
BOT2-Scores	−0.27 (0.13)	−0.13 (0.48)	−0.41 (0.02)	−0.51 (0.00)	0.56 (0.00)	
KABC-II Scores	−0.54 (0.00)	−0.58 (0.00)	−0.61 (0.00)	−0.39 (0.03)	0.35 (0.04)	0.64 (0.00)

Of all the forms of isoprostanes, only 8,12-iso-iPF2**α**-VI isoprostane had serum concentrations that consistently and negatively correlated with concentrations of serum albumin and neurology proficiency scores. Concentrations of serum albumin positively correlated with neurology proficiency scores in contrast to the trend for concentrations of triglycerides that negatively correlated with proficiency scores.

Status-specific analyses revealed that significant negative correlations between concentrations of 8,12-iso-iPF2α-VI isoprostane and neurocognitive proficiency scores were restricted to the children with konzo (all p<0.01) (**Figure 2**).

### Association models for the prediction of motor or cognitive performance

Simple (unadjusted) models showed that low serum albumin and high concentrations of triglycerides were risk factors for poor motor and cognition performances (p<0.05). Older children tend to perform poorly at cognition testing (p<0.01). High concentrations of isoprostanes, irrespective of the isomers, were associated with poor KABC-II score (p≤0.05). The duration of konzo did not correlate with the concentrations of isoprostanes (overall p>016).

After adjusting for the KABC-II score, the best overall predictive model BOT-2 score retained the concentrations of albumin and 8,12-iso-iPF2**α**-VI isoprostane as significant predictors of motor proficiency (**Table 5**).

**Table 5:** Overall association between various characteristics of interest and mean BOT-2 score, with and without adjustment for age and sex (N = 37) and concentrations of each of the biochemical predictors (N = 32).

		Model 1	Model 2	Model 3
Variable	Crude estimates (95% CI)	Adj.* estimates (95% CI)	Adj.* estimates (95% CI)	Adj.* estimates (95% CI)
Age, Years, per 1 year increase	−0.44 (−1.42, 0.53)	0.43 (−0.56, 1.42)	0.52 (−0.42, 1.45)	0.42 (−0.47, 1.32)
Gender				
Male	Reference	Reference	Reference	Reference
Female	−1.65 (−7.56, 4.26)	1.59 (−3.83, 7.02)	1.78 (−3.34, 6.91)	1.17 (−3.77, 6.11)
KABC-II score (#), per 1 unit increase	0.65 (0.37, 0.93)	0.61 (0.14, 1.07)	0.70 (0.24, 1.15)	0.82 (0.36, 1.28)
Albumin (µmol/L), per 10 µmol/l increase	0.37 (0.14, 0.61)	0.18 (−0.08, 0.44)	0.16 (−0.08, 0.41)	0.28 (0.02, 0.53)
Triglycerides (mmol/l), per 0.2 unit increase	−0.64 (−1.27, −0.01)	−0.19 (−0.78, 0.39)	−0.51 (−1.15, 0.12)	−0.54 (−1.14, 0.06)
Isoprostane III (ng/ml)	−3.16 (−25.64, 19.30)		20.77 (0.09, 41.44)	
Isoprostane VI (ng/ml)	−6.52 (−19.60, 6.54)			
8,12-iso-iPF2**α**-VI isoprostane (ng/ml)	−10.88 (−24.95, 3.20)			20.61 (4.03, 37.19)
R-square (%)		47.08%	54.81%	58.07%
BIC (lower is better)		227 (6 df)	225 (7 df)	223 (7 df)

All multivariable models include age and gender as control variables. (*)  =  Adjusted for individual predictors, (#)  =  higher score indicates greater cognition performance. Model 1  =  baseline multivariable model. Model 2  =  Model 1 supplemented with isoprostane-III. Model 3  =  Model 1 supplemented with 8,12-iso-iPF2**α**-VI isoprostane as predictor. The addition of isoprostane VI did not improve the baseline model.

After adjustment for each of the predictors including cognitive performance, only concentrations of albumin and 8,12-iso-iPF2**α**-VI isoprostane, among the biochemical measures, were significant predictors in the model explaining the largest variation (Model 3) in the BOT-2 scores; models built for all subjects irrespective of the konzo status.

After adjusting for motor proficiency, the best prediction of KABC-II score was achieved with age, gender, and 8,12-iso-iPF2**α**-VI isoprostane (**Table 6**).

**Table 6:** Overall association between various characteristics of interest and mean KABC-II score, with and without adjustment for age and sex (N = 37) and concentrations of each of the biochemical predictors (N = 32).

		Model 1	Model 2	Model 3
Variable	Crude estimates (95% CI)	Adj.* estimates (95% CI)	Adj.* estimates (95% CI)	Adj.* estimates (95% CI)
Age, Years, per 1 year increase	−1.55 (−2.44, −0.657)	−0.78 (−1.36, −0.21)	−0.65 (−1.18, −0.12)	−0.66 (−1.19, −0.13)
Gender				
Male	Reference	Reference	Reference	Reference
Female	−4.55 (−10.6, 1.47)	−2.60 (−6.02, 0.81)	−11.48 (−18.99, −3.96)	−11.1 (−18.8, −3.51)
BOT-2 score (#), per 1 unit increase	0.65 (0.37, 0.93)	0.42 (0.19, 0.66)	0.44 (0.23, 0.64)	0.46 (0.24, 0.68)
Albumin (µmol/L), per 10 µmol/l increase	0.25 (0.03, 0.48)	−0.01 (−0.21, 0.19)	−0.10 (−0.30, 0.09)	−0.11 (−0.31, 0.09)
Triglycerides (mmol/l), per 0.2 unit increase	−0.66 (−1.22, −0.10)	1.16 (−1.12, 3.44)		0.16 (−0.26, 0.57)
8,12-iso-iPF2**α**-VI isoprostane (ng/ml)	−22.56 (−32.79, −12.33)	−17.48 (−28.82, −6.14)	−33.53 (−50.61, −16.45)	−34.81 (−52.38, −17.23)
Interaction gender-8,12-iso-iPF2**α**-VI isoprostane (ng/ml)			22.37 (5.13, 39.61)	21.42 (3.83, 39.00)
R-square (%)		73.30%	78.32%	78.87%
BIC		202 (7 df)	195 (7 df)	198 (8 df)

All multivariable models include age and gender as control variables. (*)  =  Adjusted for individual predictors, (#)  =  higher score indicates greater motor proficiency. Model 1  =  baseline multivariable model. Model 2  =  Model 1 supplemented with 8,12-iso-iPF2**α**-VI isoprostane but not adjusted for concentrations of triglycerides. Model 3  =  Model 2 adjusted for concentrations of triglycerides.

After adjustment for each of the predictors including motor proficiency, only age, gender, and concentrations of 8,12-iso-iPF2**α**-VI isoprostane, among the biochemical measures, were significant predictors in the model explaining the largest variation (Model 3) in the KABC-II scores; models built for all subjects irrespective of the konzo status.

After further adjustment for case status (confounding variable in the models), only serum 8,12-iso-iPF2**α**-VI isoprostane (ng/ml), among the biochemical measures, was significantly associated with the overall cognitive performance (mean KABC-II score) (β = −32.36; 95% CI: −51.59 to −13.03; *p*<0.001) in the konzo group. Females appeared to be at higher risk and there was a significant interaction between gender and concentrations of 8,12-iso-iPF2**α**-VI isoprostane (ng/ml) (*p* = 0.05 for the interaction term). This regression model explained over 85% of the variation in the overall KABC-II score, but was not, however, significant in explaining the overall motor proficiency impairment (**Table 7**).

**Table 7:** Status-specific associations between BOT-2 or KABC-II scores and biochemical predictors, with adjustment for age, sex, and all listed terms including 8,12-iso-iPF2α-VI isoprostane.

	BOT-2 Scores		KABC-II Scores	
	Non-Konzo (N = 12)	Konzo (N = 20)	Non-Konzo (N = 12)	Konzo (N = 20)
Predictors	Estimates (95% CI)	Estimates (95% CI)	Estimates (95% CI)	Estimates (95% CI)
Age, Years, per 1 year increase	−0.83 (−0.3.37, 1.71)	0.07 (−0.17, 0.32)	−0.74 (−3.63, 2.15)	−0.46 (−1.06, 0.12)
Gender				
Male	Reference	Reference	Reference	Reference
Female	2.76 (−13.81, 19.33)	−0.52 (−1.91, 0.87)	−3.2 (−21.59, 15.14)	−10.50 (−20.4, −0.60)
KABC-II score (#), per 1 unit increase	0.49 (−0.37, 1.36)	0.18 (0.019, 0.35)		
BOT-2 score (#), per 1 unit increase			0.61 (−0.46, 1.67)	1.06 (−0.36, 2.49)
Albumin (µmol/l), per 10 units increase	0.26 (−0.77, 1.29)	0.01 (−0.06, 0.08)	0.00 (−1.19, 1.19)	−0.08 (−0.28, 0.12)
Triglycerides (mmol/l), per 0.2 unit increase	−3.57 (−13.86, 6.71)	−0.06 (−0.20, 0.09)	1.12 (−11.09, 13.36)	0.10 (−0.28, 0.49)
8,12-iso-iPF2**α**-VI isoprostane (ng/ml)	22.66 (−43.09, 88.42)	2.43 (−2.14, 7.02)	−29.97 (−100.56, 40.61)	−32.36 (−51.59, −13.02)
Gender*8,12-iso-iPF2**α**-VI isoprostane (ng/ml)				20.03 (0.77, 39.30)
R-square (%)		50.80%		85.37%

Models built for non-konzo vs. konzo children based on Model 3 from the overall assessment of associations (BOT-2 models in Table 5; KABC-II models in Table 6). (#)  =  Higher score indicates greater performance. (*)  =  Interaction term.

Status (konzo vs. non-konzo) specific models revealed that concentrations of 8,12-iso-iPF2**α**-VI isoprostane, among the biochemical measures, were the main and significant predictors in the model explaining the largest variation (Model 3) in the KABC-II scores after adjustment for motor proficiency. Association models for BOT-2 scores were not significant with the same demographic and biochemical predictors.

## Discussion

For the first time, we report the concentrations of serum F2-Isops in Congolese children and suggest a possible role of oxidative damage in the pathogenesis of cassava-associated cognition deficits. We have made every attempt to minimize artifactual changes in levels of isoprotanes by maintaining a uniform protocol across the different subjects from the time of sample collection, storage in liquid nitrogen and then −80°C, and laboratory analysis (use of BHT prior to the laboratory handling of specimens for the specific analyses of isoprostanes) [30]; performing regression analyses that adjusted for possible confounders including the levels of triglycerides; and carefully conducting internal (within group) comparisons of data from subjects who have been subjects to the same study conditions. A critical analysis of the final results showed consistency, internal and external validity of findings, and biological plausibility with regards to the potential toxic effects of cyanide i.e. oxidative stress. Despite the lack of reference values of F2-isoprostanes in healthy Congolese children, our findings retain a significant degree of relevance to the pathogenesis of konzo for several raisons. First, most of the children from Kahemba rely of improperly processed cyanogenic cassava as a main source of food and present with chronic malnutrition, all conditions that may conceivably increase risk for oxidative damage [5], [14]–[16]. Second, our study children displayed serum concentrations of F2-isoprostanes ranging from 10 to 100-times higher than those found in normal adult populations, suggesting ROS-mediated damage [19], [31], [32]. This interpretation requires, however, some degree of caution because of the lack of reference values for the African population of interest. Third, only one isomer of F2-Isops, namely the 8,12-iso-iPF2**α**-VI isoprostane, among all the studied forms, consistently showed a significant association with cognition deficits and more interestingly, only in children with the overt form of paralysis known as konzo. Last, the association between concentrations of 8,12-iso-iPF2**α**-VI isoprostane and cognition performance remained significant after adjusting for key variables such as age, gender, motor proficiency, and serum albumin as putative markers of chronic undernutrition as well as lipids (including triglycerides), precursors of F2-isoprostanes.

Both konzo and non-konzo children were exposed to cyanogenic compounds. Those with the severe form of the disease had higher concentrations of plasma SCN adjusted for concentrations of albumin suggesting a relationship between levels of cyanide exposure and motor impairment. The lack of association between concentrations of serum 8,12-iso-iPF2**α**-VI isoprostane level and motor proficiency in the regression analysis may be ascribed to our small sample size. Although the BOT-2 scores negatively correlate with serum concentrations of 8,12-iso-iPF2**α**-VI isoprostane using the Spearman crude correlation analysis, failing to demonstrate a significant association between concentrations of serum 8,12-iso-iPF2**α**-VI isoprostane level and motor proficiency in regression models may also suggest that cognitive and motor deficits associated with cassava cyanogenic poisoning do not share common susceptibility mechanisms. This proposal is consistent with previous experimental studies that showed motor and cognition differential patterns of susceptibility to cyanide poisoning [33]. It is also possible that while the BOT-2 scores reflect a motor performance that is residual to a one-time and irreversible hit on the motor system several months prior to the study, the KABC-II deficits may reflect an ongoing neuropathological process possibly captured at the time of the study i.e. concomitant to the blood sampling. Whether the deficits in cognition performance are reversible, in contrast to the well-known irreversible properties of konzo-related motor deficits, is unknown and warrants follow-up studies. Follow-up studies should also include measures of the blood cyanide concentration, another limitation of our studies, in order to better ascertain the relationship between cyanide exposure and oxidative stress injury.

The hypothesis that oxidative damage is a critical pathogenic factor suggests that other risks, notably of vascular origin, may be associated with cassava toxicity effects, particularly, under conditions of chronic malnutrition. It is therefore possible that minimal neurocognition deficits may be prevalent across the lifespan of populations that rely on toxic cassava as a main food source. The deficits may be neurodevelopmental as well as age-related, an indication for further studies on cognition in adult populations from konzo areas. The existence of cognition deficits in adults, e.g. premature age-related cognitive decline, would be a dramatic public health concern. Such studies will also help determine the diagnostic value of isoprostanes in the process of aging.

The lack of significant correlation between concentrations of urinary SCN and extent of neurological deficits as quantitatively measured by BOT-2 or KABC-II, or concentrations of F2-isoprostane isomers, is not intriguing since concentrations of metabolites of cyanide are determined by multiple factors, which may not have been controlled for in the present analysis. Furthermore, it is known that one-time on-spot urinary concentrations of SCN are poor markers of chronic cyanide poisoning, thus not suitable for phenotypic anchoring in chronic cyanide toxicity paradigms. The negative correlation between serum concentrations of albumin and those of triglycerides may be due to a metabolic adaptation following chronic dietary reliance on protein-deficient but carbohydrate-enriched cassava meals. Interestingly, the association between concentrations of 8,12-iso-iPF2**α**-VI isoprostane and cognition performance scores remained significant after adjusting for both serum albumin and triglycerides.

We conclude that 8,12-iso-iPF2**α**-VI isoprostane may be a good marker of the neuropathogenic mechanisms mediating brain injury in konzo. Whether motor and cognition deficits associated with the toxic effects of cyanide share common mechanisms and whether the later are reversible and may run across the lifespan of cassava-reliant populations, has yet to be established. The identification of markers of disease process including concentrations of F2-isoprostanes is, among other important research steps, critical for the development of interventional studies to prevent and/or reverse the neurotoxic effects of cyanogenic cassava. Interventional trials may primarily include methods for proper cassava processing prior to human consumption [34], adoption of balanced diet programs, promotion of neutraceutials i.e. foods with health benefits, as well as supplementation with antioxidants.
